# Implementation of the MiNDToolkit intervention for the management of behavioral symptoms in MND by healthcare professionals: a mixed-methods process evaluation

**DOI:** 10.1080/21678421.2024.2349924

**Published:** 2024-05-15

**Authors:** T. Katangwe-Chigamba, E. Flanagan, E. Mioshi

**Affiliations:** 1 Norwich Clinical Trials Unit, Norwich Medical School, University of East Anglia, Norwich, England and; 2 School of Health Sciences, University of East Anglia, Norwich, England

**Keywords:** Amyotrophic lateral sclerosis, motor neurone disease, carer, caregiver, trial, behavioral symptoms, ALSFTD, feasibility, process evaluation

## Abstract

**Objective:**

MiNDToolkit is a novel psychoeducational intervention for carers to support management of behavioral symptoms in people living with motor neuron disease (PlwMND). Implementation of MiNDToolkit involves delivery of an online intervention to carers, which is reinforced by trained healthcare professionals (HCPs).

**Methods:**

A mixed-methods process evaluation of the MiNDToolkit feasibility trial was conducted, focusing on reinforcement of the intervention by HCPs. Quantitative data, descriptively analyzed, were included from platform analytics, questionnaire, and 10 semi-structured interviews with HCPs. Interviews were transcribed verbatim; data were inductively analyzed using Reflective Thematic Analysis.

**Results:**

The MiNDToolkit training and platform is a beneficial and acceptable resource for HCPs with potential to increase knowledge and confidence in identifying and managing behavioral symptoms in MND. Implementation barriers included HCPs’ perceptions that highlighting behavior changes would be burdensome to carers and assumptions that carers would take the initiative to ask for support from clinicians. Degree of intervention reinforcement varied, with most HCPs delegating intervention delivery solely to the online platform.

**Conclusions:**

Implementation of the MiNDToolkit was viewed to be feasible and the platform thought to increase accessibility of support to carers. The flexible approach to delivery (online platform and optional HCP reinforcement) is acceptable as an intervention for supporting carers of PlwMND with behavioral symptoms. However, MiNDToolkit should not negate HCP involvement in providing medical and practical information to PlwMND and families. Future research should explore ways to incorporate support for carers in the management of PlwMND alongside standard care, alongside tools such as the MiNDToolkit.

## Background

Evidence of cognitive and behavioral symptoms in MND has grown significantly in recent years (1, 2). Symptoms include apathy, impulsivity, ridigity, executive and language dysfunction (2). Detection of these symptoms has also improved, with many cognitive and/or behavioral screening tests now available (3, 4). These assessments have been operationalized in the updated diagnostic criteria for ALSFTD) (5), easing clinical detection and research studies.

Healthcare professionals (HCPs) can administer cognitive and behavioral assessments (3). However, despite availability, services do not use the assessments regularly, or use them reactively, e.g. when severe symptoms are observed. Moreover, even when assessments for people living with MND (PlwMND) are done systematically, there are no guidelines on the management of cognitive and behavioral symptoms (6). This is a concern that does not go unnoticed: at least 50% of PlwMND present with cognitive and behavioral symptoms, and these can affect survival rates (7–9).

Recently, initiatives to address this evidence gap have started to surface. A peer-support group delivered online to small groups of carers was tested in Denmark (10), with results demonstrating that carers engaged well with online interactions. More recently, the MiNDToolkit emerged as a new psychoeducational intervention for carers to support the management of behavioral symptoms, delivered by a bespoke online platform and reinforced by trained HCPs. The recently completed feasibility clinical trial (11) showed the MiNDToolkit to be feasible and warranted further evaluation of its efficacy and effectiveness.

**Figure 1.**
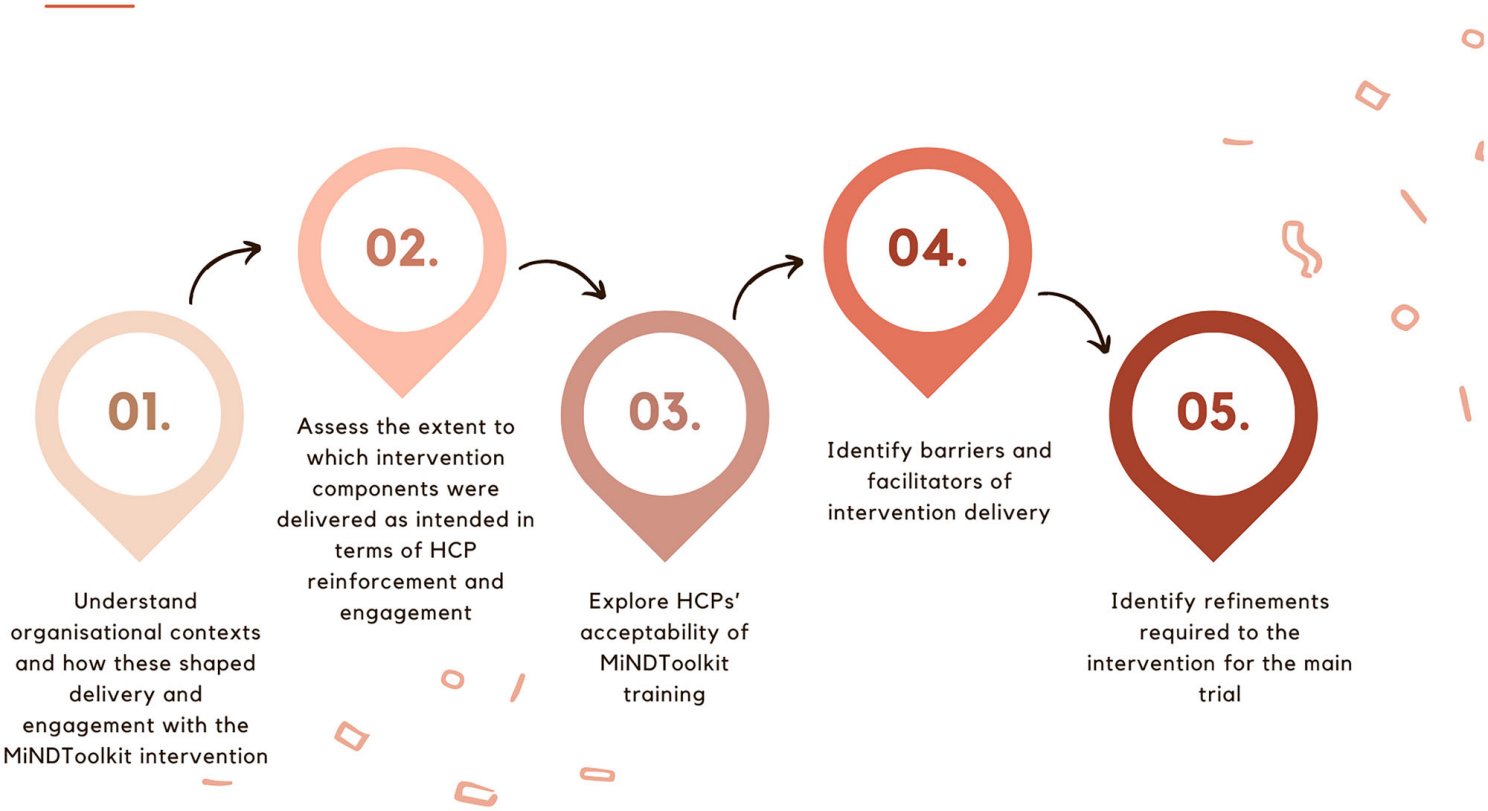
MiNDToolkit mixed methods process evaluation objectives.

In this nested mixed-methods process evaluation, we set out to understand the experiences of HCPs in managing behavioral symptoms before MiNDToolkit, and their experiences using the platform, including training. Carers’ experiences of using MiNDToolkit are reported separately (12). This study’s aims were to (i) assess implementation of the MiNDToolkit, (ii) understand contextual factors shaping intervention delivery, and (iii) investigate how to refine and optimize the intervention delivery (Figure 1).

## Methods

### Design

A mixed-methods process evaluation including intervention platform analytics, questionnaires, and semi-structured interviews was conducted. The design, conduct and reporting of the study was informed by the UK-Medical Research Council guidance on process evaluation of complex interventions (13).

### Study setting

The MiNDToolkit study (ISRCTN 15746123) was a randomized controlled trial to determine feasibility of the MiNDToolkit for use by carers with optional support from HCPs. The study was conducted across 11 sites in England and Wales between July/2021-March/2023. Sites varied but all had advanced MND care set up.

### The MiNDToolkit

MiNDToolkit is a complex intervention (11) for the management of behavioral symptoms. In brief, carers access tailored online modules to manage symptoms reported on screening questionnaires also collected via the platform. Trained HCPs reinforce the learning and strategies during appointments. Training for HCPs includes a 2-hour online session, a 90-min group training session with role playing, and optional weekly supervisions throughout the trial.

### Participants and recruitment

27 HCPs from participating sites were eligible to take part in this study. HCPs were purposively sampled to achieve maximum variation of site, professional role, engagement with the MiNDToolkit platform and reinforcement of the intervention.

### Data collection

Ten individual semi-structured interviews were conducted. HCPs were asked about using the platform, reinforcing strategies with carers, perceived barriers/enablers to implementation, and proposed changes. To further explore engagement and experiences with training and implementation, interview data were triangulated with a questionnaire completed by HCPs before and after the training, and platform analytics - frequency of use and reinforcement notes.

Interviews were conducted virtually (TKC) on MS Teams, audio recorded, and professionally transcribed verbatim (Topic guide, Appendix-1).

### Analysis

Quantitative data were descriptively analyzed. Interview data were inductively analyzed using Reflective Thematic Analysis (14) to understand how training was experienced, how MiNDToolkit was implemented and how service structures shaped delivery; NVivo v.12 was used (QSR). More details on Appendix-2.

## Results

### Context

#### Services’ structure for managing behavioral symptoms in MND and ALSFTD

In England and Wales, services are commissioned locally through integrated care systems (ICSs), which are partnerships of organizations responsible for planning and delivering healthcare services. Characteristics of HCPs participating in the process evaluation, profession and sites are presented in Table 1; these represented 10/27 HCPs involved in the trial (11).

**Table 1.** Characteristics of HCPs interviewed for the process evaluation of the MiNDToolkit feasibility study.

HCP ID and Site reference	Professional background	Type of service
HCP_01, Site A	Neuropsychologist	Specialist MND Care Center
HCP_02, Site B	Occupational therapist	Community Rehabilitation Team
HCP_03, Site B	Occupational therapist	Community Rehabilitation Team
HCP_04, Site C	Palliative Care Consultant (medic)	Hospice
HCP_05, Site D	Occupational therapist	Community Trust
HCP_06, Site E	Nurse	Secondary Hospital
HCP_07, Site D	Speech and language therapist	Community Trust
HCP_08, Site F	Nurse	Hospice
HCP_09, Site G	Nurse	Specialist MND Care Center
HCP_10, Site H	Nurse	Specialist MND Care Center

Types of services offered to PlwMND experiencing behavioral symptoms prior to the MiNDToolkit varied greatly by sites. Although most teams were aware of the ECAS(3) assessment to support diagnosis of cognitive and behavioral symptoms, services administered it sporadically; a few offered it routinely. Moreover, due to lack of follow-on support, some HCPs expressed reluctance in offering the Edinburgh Cognitive and Behavioural ALS Screen - ECAS (Table 2, quote-A).

**Table 2.** MiNDToolkit process evaluation themes, subthemes and quotes.

1	**Context**	1.1	Services’ structure for managing behavioral symptoms in MND and MNDFTD	A	“We had started to do the cognitive assessment screen, the ECAS, but we didn’t do it reliably, we did it if we suspected something, it was very hit and miss really and we didn’t really have a plan of what we put into place following on from that, you know, how we helped the family or how we helped the person” **HCP 02, occupational therapist**
				B	“When there’s evidence of behavioral change, I’m not able to see carers on their own because of the way the hospital, just the way things are set up where I work, but I tend to do it jointly with the person living with MND…and have the carer very involved in those sessions with the person living with MND’s consent. But sometimes it’s difficult to cover everything or sometimes the person living with MND doesn’t want to participate in those kinds of sessions.” **HCP 01, neuropsychologist**
		1.2	Healthcare Professionals’ approach to management of behavioral symptoms of MND and MNDFTD prior to the MiNDToolkit study	C	“It’s almost like they [HCPs] can cope with the physical side but as soon as there’s any change cognitively or behaviorally, everybody sort of throws their hands up in a panic and isn’t really sure what to do. So, I think people are scared of that side of things and that’s why I wanted to be involved in the toolkit because I don’t feel, even with my dementia background, I didn’t have the tools to use” **HCP 10, nurse**
	**Implementation**	2.1	Acceptability and training experience	D	“For me that was the best thing about the toolkit, was the training that myself and my colleagues received. And I would love that to be available for everybody, whether they did the toolkit or not, because it was so helpful, it was so in-depth…it is the most comprehensive training package I’ve seen on that topic. So, I think it would be amazing for it to be available for everybody in an educational session. I would say everybody that would be involved in looking after patients and carers with MND. So we have 12 clinics in our patch and they are consultants, palliative care consultants, who know as much as other people about, you know, behavioral and personality change. I don’t think anybody is really specialist in that, in the coping strategies and all of that sort of thing” **HCP 10, nurse**
				E	"I was really lucky, it was just myself and another trainee, so there was two of us, it was quite an intimate group. So, it was really good to be able to have proper discussions and get feedback straightaway. So we would answer the case scenarios questions and try and decide which resource we were going to use and which approach we’re going to use for that particular scenario…I felt like having those really close knit discussions allowed us to just have a go and if it wasn’t right then we had a good discussion around why you wouldn’t use that particular resource and you would use this instead**" HCP 07, speech and language therapist**
2				F	“I think it was really useful, like the drop-ins and if you think about the sort of environment at the moment in terms of people being out of time for study leave and doing things like this, I think it definitely needs to be as flexible as it can be because people are having to fight and negotiate very hard to get, you know, time away from clinical work to do things. So that’s where that kind of fairly open access to the team for support, I know that comes at a cost, but it’s probably a useful investment in terms of getting people engaged and actually coming together with other sort of PI’s and things doing the project” **HCP 09, nurse**
	**Implementation**	2.2	Training outcomes: increased knowledge and confidence	G	“It definitely opened my eyes in terms of when the person is presenting in a certain way, what we can do to support that particular area of like behavioral difficulty or whatnot. It definitely gave me a resource as a therapist to feel confident about as well because actually the wording in a lot of the resources were very generic for – and you can adapt them for certain patients and carers. Yeah, I found the resources really helped prepare me. And also going into my sessions, I knew I had those tools, so I didn’t feel like I needed to spend too much time preparing because it was about the discussion that was happening in that session and then I could just refer to whichever resource I needed…And it was always linking the behavior with the disease which made my carer feel much more at ease and comfortable with because it was always reiterating that it was not personal and it was to do with the disease and this was a symptom of the disease and this is how we can help as, you know, to manage it, so” **HCP 07, speech and language therapist**
2		2.3	Recruitment: identifying eligible carers and offering them the MiNDToolkit	H	"And the way that the study team had suggested… from when we started recruitment, we were encouraged to recruit any patient with MND, not just the patients who we believed had behavioral issues, which seemed a bit odd when we were talking about a study about helping carers manage behavioral issues of MND. But it was sometimes quite a good way to talk about, you know, the fact that we do see behavioral issues in a fair proportion of patients with MND" **HCP 04, palliative care consultant**
				I	“So, the way it’s been embedded in, for the people that I see, which is primarily through clinics…felt that that wasn’t a great way to embed it, we weren’t getting masses of interest and we sort of took a slightly more proactive approach by writing to people directly, which I felt a bit uncomfortable with to start with…that was quite a big issue for me in terms of feeling anxious… But in terms of recruitment that was quite a turning point…it was almost like people received it and very quickly thought, “Ah yes, this is me.” And that was important because we perhaps wouldn’t have known people without the right questions and that they were experiencing most difficulties.…we went from really struggling to suddenly fulfilling our target numbers very quickly” **HCP 09, nurse**
				J	“As a family they would really benefit from the support but obviously the first language isn’t English and I don’t know how transferable the toolkit would be into other formats…We did invite them because it’s very reliant on English speaking members of the family to then do it but they’re the ones that are younger and working and busy and actually it’s his wife that needs it all and she doesn’t speak any English and possibly isn’t IT literate either. So I think the two barriers for me would possibly be the language and possibly for the people that are a little bit older or not IT literate or don’t have access to [the internet]…If I could have given her a booklet, for example, in Urdu which she might have sat and read some of it and taken it on board possibly" **HCP 03, occupational therapist**
2	**Implementation**	2.4	Reinforcing strategies to carers during visits or consultations.	K	“So the first person, who was [Carer name], was very interested in it and she completed it quite quickly and she’s the one who said [had] a light bulb moment…she really loved it. She just found the information really useful and…she said, it was going to change how she responded to him, and she didn’t really feel like she needed me to go in there and problem solve with her and talk about strategies, she just didn’t need it” **HCP 02, occupational therapist**
				L	“When it started they sort of said, “You don’t need to see them anymore than you normally see them” But then in my job as the MND Care Coordinator, I might not see somebody for three months, they’d be off the study by then…then it sort of changed a little bit [because they were] trying to work out how much [reinforcement] makes a difference, so some people would be seeing quite a lot of them. And I found that a little bit confusing. [For] the next part of the study, I think it probably would be better to say “We would like you to follow this up on a monthly basis or a fortnightly basis,”that would be helpful to have a guide because when you’re busy and you think, “Well I don’t actually have to do them any more than I normally would” **HCP 02, occupational therapist**
		2.5	Platform engagement	M	“To be honest when the MND Specialist Nurse is going in…she was reading more my notes…and yeah, in the session she would touch on the MiNDToolkit. There’s lots of other things to do when we’re in with them, besides the MiNDToolkit too, so it’s choosing what to do first or just using the toolkit resources throughout the session while you’re talking about whatever else you’re doing, which you can easily do too. But I think the MND Specialist Nurse didn’t have as many notes…, but she did say to me that it was very helpful to see my notes, to see before she went in, what to expect and what sort of types of ways I was reinforcing and how the carer was responding to that” **HCP 07, speech and language therapist**

Apart from use of medication, most services offered no support for the management of cognitive and behavioral symptoms. Few services offered advice from a psychologist; generally, HCPs viewed the management of cognitive and behavioral symptoms as a psychologist’s speciality. As such, sites without psychological expertise either had no follow-on support, or co-ordinated with other teams to provide support, e.g. Memory Clinics. The lack of standardized support for cognitive and behavioral symptoms was a key reason why HCPs signed up for the MiNDToolkit trial.

*I’ve worked probably the best part of 12 years directly with people with MND […] it’s an area that I […] felt possibly less well equipped to help manage [HCP 09, Nurse].*

Sites offered very limited support for carers. Additionally, the current service structure, which solely focuses on patients, was highlighted as a barrier to supporting carers, which resonated with experiences of carers (Table 2, quote-B) (12).

#### HCPs’ approach to management of behavioral symptoms of MND and ALSFTD prior to the MiNDToolkit trial

Most services did not have an approach to managing behavioral symptoms prior to the MiNDToolkit study, except for sites A and F.

*There wasn’t any support because we weren’t really recognising it […] and on top of that you haven’t really got a plan of how you help them [HCP 02, Occupational Therapist].*

HCPs’ lack of confidence or expertise in identifying and managing behavioral symptoms prior to MiNDToolkit was highlighted as a source of anxiety, often leading to their own behavioral avoidance and potentially incorrect assumptions. For example, some HCPs felt raising the topic with carers too difficult, expressing their helplessness as they felt behavioral symptoms were “another thing” in the management of MND that they could not do anything about. Others felt that discussing behavioral symptoms would cause additional concerns and burden on families, which is an assumption not shared by most carers (12). (Table 2, quote-C).

*I don’t think we brought it up because it’s quite difficult to talk about… and you can justify that, well you know, there’s so much going on for the person with MND [HCP 02, Occupational Therapist].*

Some HCPs presumed that behavioral symptoms were not a priority for carers. Additionally, despite HCPs acknowledging that some carers might be hesitant to speak up about behavioral symptoms, there was an assumption that carers faced with symptoms would be able to identify and name them on their own, and take the initiative to reach out to HCPs when needed.

### Implementation

#### Acceptability and training experience

The training offered useful learning for all HCPs, regardless of their level of expertise and the platform was highlighted as a good educational resource (Table 2, quote-D). HCPs expressed the importance in recognizing and understanding behavioral symptoms and developing expertise to support and validate these experiences to PlwMND and their families. Indeed, validation of behavioral symptoms was a key pillar of the MiNDToolkit training.

Due to their perceived limited time, HCPs felt having a platform was crucial for providing specialist support, addressing important topics for carers. HCPs found it useful that modules were tailored to the PlwMND’s symptoms, providing a resource that explained most relevant symptoms for carers and offered simple and practical strategies. HCPs felt that having symptoms explained to be part of MND was most important for allowing families to reconcile challenging behaviors at a time when they would like to make the most of their time together.

*A lot of relatives and carers struggle with apathy because they think, if you’ve got a short life span, let’s do this, let’s do that […] and when their loved one doesn’t want to, they can’t understand it […] So for them to understand that it’s part of the disease is really important. And I don’t think we were very good at [explaining] that before and the platform explains that behaviour is caused by this [HCP 06, Nurse].*

The structure of the training, consisting of 1) platform bite-size modules, 2) online training session with the Chief Investigator (EM), 3) platform resources, and 4) drop-in supervision sessions, was acceptable to HCPs. Some HCPs completed online training outside of their office hours. HCPs highlighted the virtual face-to-face component as most important, allowing them to have discussions about how to approach different scenarios and offering opportunity to practise these. HCPs expressed need to dedicate more time for this session in future, preferably in smaller groups, to ensure adequate opportunity for in-depth discussions and questions (Table 2, quote-E). Additionally, despite HCPs finding the drop-in session useful, uptake was low (3/27, on average). Those joining drop-in sessions were very engaged with the study (Table 2, quote-F).

Another suggestion was to offer online videos of real-life consultations demonstrating good communication skills. This stemmed from one HCP feeling very anxious about role playing in front of colleagues.

#### Training outcomes: increased knowledge and confidence

Despite diverse levels of knowledge/experience in the management of behavioral symptoms, most HCPs felt the platform was an invaluable educational resource for practitioners. HCPs felt that the training had given them a thorough background understanding of behavioral symptoms in the ALSFTD spectrum and provided skills and knowledge to identify the symptoms. Additionally, HCPs felt that they had received clear examples of when to introduce and apply suggested practical strategies to manage these symptoms.

*I did find it all really helpful […] for example, apathy and understanding how it might be seen to be depression and how you might distinguish which was going on [HCP 09, Nurse].*

HCPs’ knowledge before and after the training was assessed with a quiz. HCPs’ knowledge (all 27 HCPs) pretest mean was 66.67% (95%CI: 60.39 to 72.94). The post-training quiz showed that HCPs’ mean score was 79.73% (95%CI: 73.11 to 84.36). Overall, 23/27 HCPs improved their quiz scores (increases 1–5 points). Only one HCP, not interviewed, already scored 100% at the pre-training phase. The change in knowledge scores confirmed HCPs’ reflections in interviews, with some expressing surprise at how much they did not know about behavioral symptoms and felt that the training provided them with a much-needed opportunity to address this gap.

*I thought I knew quite a bit about it but it turns out I didn’t, I learnt a lot from doing the training [HCP 06, Nurse].*

HCPs also shared how training had increased their confidence to have conversations with carers about behavioral symptoms. Additionally, they felt that the platform offered the right tools and resources to address the topic, including strategies during consultations (Table 2, quote-G).

#### Recruitment: identifying eligible carers and offering MiNDToolkit

Most HCPs had no previous research experience, with some taking on the role of Principal Investigator (PI) for the first time. HCPs’ role in recruitment was to offer the study to carers, following which screening for eligibility would be undertaken via the platform. This approach was viewed favorably as it increased HCP capacity to recruit carers without undertaking the clinical screening. It also enabled dissemination of information to all carers in their caseload (Table 2, quote-H).

*And I think that element of we didn’t do the screening, you did it, enabled us to offer it to loads and loads of people [HCP 06, Nurse].*

However, the initial approach to recruitment taken by most sites was to offer the MiNDToolkit only face-to-face. On reflection, HCPs felt this approach led to low recruitment rates early in the study. In later stages of recruitment, HCPs worked with the research team to adopt more proactive recruitment, offering MiNDToolkit via letters or phone calls – which contributed to the study reaching its recruitment target (Table 2, quote-I).

HCPs highlighted the importance of offering the intervention early to maximize benefits for carers. HCPs’ perceptions of key determinants of carers uptake and engagement with MiNDToolkit included: carers’ capacity amidst responsibilities, carers’ readiness to learn more about behavioral symptoms, language (non-English speakers) and digital literacy. Although these perceived determinants were applicable to some carers, HCPs who offered MiNDToolkit despite their perceptions were often surprised at the positive response from carers (Table 2, quote-J):

*I offered it despite thinking, “Oh, computer, tech wise she’s older, is she going to get on with it?” But then actually she was emailing me back and forth about thing [HCP 08, Nurse].*

#### Reinforcing strategies to carers during appointments

HCPs reinforcing strategies to carers varied across sites, with 5/27 HCPs providing reinforcement. Reinforcement, designed to be conducted as part of a consultation, involved HCPs and carers openly discussing management of behavioral symptoms and strategy implementation. Finding time to discuss the MiNDToolkit away from the PlwMND was a challenge, especially for HCPs working in secondary care settings and for carers who did not want to disclose to the PlwMND that they are receiving MiNDToolkit support.

*I found it more difficult with the person with MND next to them, to go into any great depth…I’m largely clinic based, it has been quite difficult to reinforce the strategies so, I haven’t engaged in that bit as well as I would have hoped to [HCP 09, Nurse].*

HCPs and carers working flexibly to discuss the content of the MiNDToolkit depended on capacity, environment (clinic vs home) and needs of the carers. As such, reinforcement interactions looked different for each carer-HCP dyad and included one-to-one discussions over the phone or at the doorstep following home visits. On reflection, most HCPs felt that the platform offered comprehensive support for carers, and that not all carers needed reinforcement. However, HCPs felt it still important to be aware of what carers know and check-in with them. Overall, HCPs who reinforced the MiNDToolkit felt that reinforcement was a motivation for carers and important for their engagement and does not take too much extra time (approx. 10min) during appointments (Table 2, quote-K).

Another key challenge highlighted by HCPs was the ambiguity in the guidance regarding reinforcement where some attempted to align reinforcement with usual appointments, which can be 3-monthly (where the feasibility trial period was approximately 3 months), whereas others kept regular contact with carers outside of these appointments. As such, HCPs expressed the need for greater clarity around frequency of reinforcement (e.g. monthly) and a structured meeting plan which might involve pre-arranging this with carers (Table 2, quote-L).

#### Platform engagement

Beyond training, the MiNDToolkit provides HCPs with resources and strategies for use during appointments. The platform is also designed to facilitate a Multidisciplinary Team (MDT) approach, providing a space to record notes and keep up-to-date with carers’ progress. As with reinforcement, use of the platform varied greatly (Table 2, quote-M).

Practically, a few HCPs experienced difficulties linking strategies to behavioral symptoms when preparing for the appointments and found the combination of strategies difficult to remember. A suggestion emerged to simplify strategies by using an acronym and/or using single words rather than word combinations. HCPs also felt that developing a paper resource of strategies which could serve as a reminder during visits.

## Future implementation

Overall implementation of MiNDToolkit was viewed to be feasible and the platform was thought to increase accessibility. For inclusivity, HCPs recommended the continuation of the hybrid delivery approach (i.e. online with HCP reinforcement), delivering the intervention in other formats (e.g. books and Apps), and adding other language options (e.g. Urdu).

The implementation of MiNDToolkit into routine practice was viewed positively by all HCPs, most of whom highlighted that there is currently no other intervention of its kind. There was willingness from some HCPs to even pay for MiNDToolkit to be implemented in their service. The MDT approach to training and delivery of the intervention was also viewed favorably by HCPs as this would ensure that families are supported holistically without depending on one HCP only.

*I don’t think there’s anything else like it in the MND… And now that [we] have done the MiNDToolkit, we are more aware when we have our meetings with patients and we had one yesterday with somebody who has got, very rigid thinking and lack of insight [HCP 02, occupational therapist]*

In some sites, use of MiNDToolkit led to wider impact for service setup. For example, the MiNDToolkit triggered the creation of a management pathway involving early identification and timely support. Previous attempts to pathway creation had been unsuccessful because MND was viewed as primarily a physical condition. Following the training, HCPs felt more confident and skilled to initiate conversations with their colleagues to create a care pathway for PlwMND experiencing behavioral symptoms and their families.

*We have standardised a template on SystmOne now with the ECAS templates - the MiNDToolkit prompted that. The other thing that’s come off the back of this, is we’ve never had a pathway with mental health services before, they will not see patients with MND because in their opinion it’s more of a […] physical condition. But we have actually met with the mental health doctors and pulled a pathway together [HCP 03, Occupational Therapist]*

## Discussion

The MiNDToolkit was recognized as a beneficial educational resource for HCPs and an acceptable intervention for supporting carers of PlwMND with behavioral symptoms. The virtual training and bespoke platform consisting of educational modules, structured interactive clinical reasoning and strategies, facilitated the increase of HCP knowledge and confidence in identifying behavioral symptoms in MND and providing appropriate advice. Against the contextual backdrop of limited HCP training and limited support for families of PlwMND, motivation to engage in training was consistently high throughout the study.

Our findings reveal that HCPs’ perceptions (e.g. mentioning behavioral symptoms would be burdensome to carers) and assumptions (e.g. carers can take initiative to ask for support from clinicians) about carers’ communication and support needs could pose as potential barriers to offering appropriate carers support. These assumptions and perceptions, which partly stemmed from HCPs’ lack of training and confidence in identifying behavioral symptoms and providing follow-on support, highlight the need for HCPs to receive specialist training. With recent evidence suggesting that PlwMND and carers rely on clinicians for medical and practical information (15), it is vital that HCPs receive appropriate training and resources for the management of behavioral symptoms. In addition, it is important that discussions on communication preferences in response to changing needs and disease progression are held early in the therapeutic relationship to eliminate assumptions which might hinder the provision of support (15).

Our findings suggest that although MiNDToolkit training has a definite role in increasing knowledge and providing necessary tools, there is still a need for HCPs to have regular use and application of the learning to gain experience and develop confidence. In the MiNDToolkit feasibility study, the lack of experience and confidence in identifying behavioral symptoms in PlwMND posed an initial barrier to recruitment. However, the platform’s inbuilt screening feature minimized the need for HCPs to make decisions around the eligibility based on experience. Moreover, the provision of specialist resources that explain symptoms most relevant for carers and provide simple and practical strategies for these symptoms was positively viewed by HCPs.

The MiNDToolkit, originally designed for delivery in person by HCPs, was subsequently re-designed for delivery via a bespoke platform and optional HCP reinforcement due to the COVID-19 pandemic. This new flexible approach to intervention delivery was positively regarded by HCPs for its potential to minimize time capacity challenges whilst still allowing for human interaction, when needed or preferred by carers (16). However, our assessment of intervention fidelity, which for HCPs consisted of platform use and reinforcement, showed that most HCPs delegated the intervention delivery primarily to the platform, and did not take ownership of the reinforcement/intervention. Findings showed that intervention setting, i.e. clinic vs home setting, played a key role in determining whether appointments allowed for the provision of both PlwMND and carer-focused support. The care of PlwMND requires significant input from carers, most of whom are informal carers. With previous research highlighting the burden, psychological and emotional impact associated with informal care of people with MND (17), there is a need to reconfigure how care is delivered to PlwMND and their families (18). Although the MiNDToolkit can serve as a standalone intervention, carers involved in the feasibility study unanimously expressed the need for HCP involvement in their support (12). Therefore, the need to further explore how best to incorporate support for carers in the management of PlwMND alongside interventions such as MiNDToolkit requires further exploration.

Finally, the feasibility of the MiNDToolkit study (11) provides a foundation that facilitates progress to the full RCT. The enthusiasm for research displayed by HCPs, several of whom were PIs for the first time, has created the potential to efficiently link services in the UK, and facilitate the progress of applied research in MND.

## Conclusion

The MiNDToolkit is a beneficial educational resource for HCPs involved in the management of behavioral symptoms in PlwMND. The flexible approach to delivery via online platform with optional reinforcement is acceptable as an intervention for carers of PlwMND displaying behavioral symptoms. The MiNDToolkit does not negate the need for HCP involvement in providing medical and practical support to PlwMND and families. As such, there is still a need to explore ways to incorporate support for carers in the management of PlwMND alongside standard care, with tools such as the MiNDToolkit.

## Supplementary Material

Supplemental Material

## Data Availability

The study data have not been made publicly available due their sensitive nature (interviews) but those interested in exploring collaborations should contact the corresponding author.
