# Supplementary material for: Implementation of the MiNDToolkit intervention for the management of behavioral symptoms in MND by healthcare professionals: a mixed-methods process evaluation
Source: Amyotroph Lateral Scler Frontotemporal Degener. 2024 May 15;25(5-6):496–505. doi: 10.1080/21678421.2024.2349924 (PMC11286209; doi:10.1080/21678421.2024.2349924)
Supplement: Supplemental Material [file IAFD_A_2349924_SM2804.zip › Appendix_1.docx]

**Appendix 1 – Interview topic guide template**

**Interview guide – MiNDToolkit**

**Interventionist: Health Care Professional (HCP)**

1. **Introduction**

Thanks. Introduce self. Re-state purpose of the interview and use of audio-recording

1. **Background**

Could you start by telling us your name, and the name of your team? Could you tell me how many other colleagues in your team were also trained and are using the MiNDToolkit?

1. **Programme experience**

Have you ever been involved with an intervention for carers of people with MND before? If so, what was it about? In what ways was it similar or different to the MiNDToolkit?

What was your experience of the MiNDToolkit Online Platform? (positive and negative features)

What was your experience with the training? (positive and negative features)

What were you hoping that carers would get out of the MiNDToolkit Online Platform? Do you think those expectations/needs were met? Why?

Were there features of the MiNDToolkit Online Platform that you think were particularly enjoyed by the carers? Or were particularly helpful for the carers? What were they and why were they enjoyable/helpful?

Were there features of the MiNDToolkit Online Platform that you think the carers did not enjoy or were not helpful? What were they and why do you think they did not work so well?

What did you think of the format of the MiNDToolkit Online Platform? (online nature of the intervention; use of automatic reminders; frequency of sessions/duration of sessions?; the modules; animations and videos)

What was your experience of having the MiNDToolkit Online Platform being offered by your MND/Community team? (Positive and negative)

Would you say that you were able to fully engage with the platform? If so, why? If not, what were the barriers?

1. **Next steps in the MiNDToolkit**

Would you want to see the MiNDToolkit Online Platform been offered to other HCPs/carers in the future? Why?

1. **Other comments**

Any other issues that the HCP would like to raise. Thank them for their time.

MiNDToolkit Topic guide v1.0 03Apr2021 IRAS 260290
