# Supplementary material for: Implementation of the MiNDToolkit intervention for the management of behavioral symptoms in MND by healthcare professionals: a mixed-methods process evaluation
Source: Amyotroph Lateral Scler Frontotemporal Degener. 2024 May 15;25(5-6):496–505. doi: 10.1080/21678421.2024.2349924 (PMC11286209; doi:10.1080/21678421.2024.2349924)
Supplement: Supplemental Material [file IAFD_A_2349924_SM2804.zip › Appendix_2.docx]

**Appendix 2– more details on methods**

Two researchers, TKC and EM, were involved in the data analysis, working collaboratively and reflectively by sense-checking ideas, and exploring multiple assumptions or interpretations of the data with the aim of achieving richer interpretations of meaning, rather than attempting to achieve consensus of meaning. TKC is a methodologist, specialising in mixed methods process evaluation. EM has extensive clinical and research experience in the MNDFTD continuum and is an occupational therapist by background.

The analytical process involved an initial familiarisation with the data, whereby the researchers firstly listened to each interview recording, making notes of initial analytic observations and insights, both in relation to each individual data and to the entire dataset. Following this, the two researchers met to discuss initial thoughts and observations about the data, starting to develop an understanding of the primary areas addressed during the interviews.

Following familiarisation, TKC then read and re-read the interview transcripts data, to become immersed and intimately familiar with its content, before coding the data by working systematically through the entire dataset to identify and label aspects that were interesting, relevant to the key objectives of the study and could be informative in developing themes. Following completion of the coding, the NVIVO folder was shared with EM who reviewed and refined the codes by sense checking them, ensuring that the labels provided sufficient detail to explain what was interpreted from the related data item. During the process EM also identified codes which were conducive to interpreting themes.

Theme generation involved the researchers meeting to discuss and examine the codes and collated data to begin to develop significant broader patterns of meaning (potential themes). During this meeting the researchers identified key themes in the data. At this stage, the researchers also identified points which needed triangulation with the quantitative data. For example, where HCPs were reporting increased knowledge about behavioural symptoms and their management following the training, this was triangulated with the knowledge questionnaire data. Final themes identified were then aligned with a priori evaluation objectives underpinned by the MRC guidance on process evaluation for reporting.
